# Peripheral Nerve Stimulation for a Refractory Case of Postherpetic Neuralgia in the Upper Limb: A Case Report

**DOI:** 10.7759/cureus.55168

**Published:** 2024-02-28

**Authors:** Arun Kalava, Simeon V Mihaylov, Harriet Kaye Austin, Saru Acharya

**Affiliations:** 1 Anesthesiology, University of Central Florida College of Medicine, Orlando, USA; 2 Pain Management, TampaPainMD, Tampa, USA; 3 Anesthesia and Critical Care, Postgraduate Institute of Medical Education and Research, Chandigarh, IND

**Keywords:** sprint pns, varicella-zoster virus, vzv, pns, phn, upper limb, peripheral nerve stimulation, postherpetic neuralgia

## Abstract

Postherpetic neuralgia (PHN) is a chronic neuropathic pain syndrome that is a direct consequence of the reactivation of varicella zoster virus (VZV). It manifests as neuropathic pain, which is pain that occurs because of dysfunction or damage of the nerves that carry sensations to the brain, and this typically persists for months to years after herpes zoster. Current conservative management for PHN includes a combination of topical agents (i.e., lidocaine and capsaicin) and systemic therapy (i.e., serotonin and norepinephrine reuptake inhibitors (SNRIs), gabapentin, pregabalin, and opioids). For refractory cases, with persistent intractable pain, more invasive interventional techniques can be used as pain-relieving measures to improve the patient’s quality of life. This report presents a patient with upper limb PHN who responded to peripheral nerve stimulation (PNS) after he failed to obtain sufficient pain relief with conservative management.

## Introduction

Postherpetic neuralgia (PHN) is a chronic neuropathic pain syndrome that is a direct consequence of the reactivation of varicella zoster virus (VZV) known as herpes zoster (HZ), colloquially known as shingles [1]. VZV is a DNA virus that tends to remain dormant in the sensory ganglia after primary infection with chickenpox during childhood. HZ manifests in those with decreased or declining immunity as a painful, vesicular rash in a dermatomal distribution [2]. As the virus travels down the sensory nerves, inflammation and damage sustained along these nerves result in neuropathic pain that persists months to years after HZ, leading to PHN that results in a significant reduction in quality of life.

Current conservative management for PHN includes a combination of topical agents (i.e., lidocaine, capsaicin, or a combination of anesthetics and analgesics such as LidoPro®) and systemic therapy (i.e., SNRIs, gabapentin, pregabalin, and opioids) [3,4]. Not only are elderly patients at increased risk of PHN, but they also tend to present with comorbid diseases, requiring multiple medications. This raises the concern for adverse effects and medication interactions during the treatment of pain. For refractory cases and continued intractable pain, more invasive interventional techniques (i.e., nerve blocks, epidural steroid injections, radiofrequency ablation, and spinal cord stimulation (SCS)) can be used as pain-relieving measures to improve the patient’s quality of life. 

This case report presents a patient with upper limb PHN who responded to peripheral nerve stimulation (PNS) after he failed to obtain sufficient pain relief with conservative management.

## Case presentation

A 65-year-old Caucasian male with a medical history significant for Sjogren’s syndrome, non-Hodgkin lymphoma, hypertension, and hypothyroidism presented with PHN involving the left upper extremity (C5-C6 nerve root distribution). The pain has persisted for four months and was 9/10 on a numerical rating scale. It prevented the patient from moving his thumb and making a fist. He also reported fatigue, trouble sleeping, and weight gain because of the pain.

The patient was 69.6 inches tall, weighed 206 lbs, with BMI of 29.9, placing him in the overweight category based on the CDC’s guidelines. A physical exam of the cervical spine was unremarkable. The skin of the left upper extremity showed no signs of rash or inflammation; however, it was significant for hypersensitivity on the dorsum of the thumb and the palmar aspect of the hand. Active and passive motion of the thumb were limited because of pain. Thenar atrophy was also noted.

Before establishing care with us, the patient had explored the following treatment modalities: conservative management consisting of pregabalin 150 mg three times a day, and methadone 5 mg twice daily, which have managed to decrease the pain from 10/10 to 9/10. Invasive treatment modalities included a cervical epidural corticosteroid injection, which resulted in temporary pain relief. A left stellate ganglion block was also done, but it provided no pain relief. The patient reported the most significant improvement in symptoms from acupuncture, which only lowered the pain score to 8/10.

When the patient presented to our clinic after four months of therapy, he continued to experience debilitating symptoms. A diagnostic left median and left superficial radial nerve blocks were performed in the office, wherein each nerve was infiltrated with 7.5 mL of ropivacaine 0.15% and 10 mg of dexamethasone. The patient reported >90% relief of pain following the block, with pain level lowering to 1/10. Therapeutic options such as oral or IV ketamine, IV lidocaine, capsaicin patch, SCS, and PNS were also discussed. The patient’s medical history of non-Hodgkin lymphoma rendered him a poor candidate for therapeutic low-level laser therapy (LLLT) because of the controversial effects of LLLT promoting cancer growth through modulation of cellular proliferation, angiogenesis, and other inflammatory metabolites [5].

The pain relief and decrease in visual analog scale (VAS) following the nerve block was sustained for two days and returned to baseline. Subsequently, an 8% capsaicin patch was applied over the forearm for one hour in the office. At his follow-up appointment, three months later, the patient reported no significant pain relief from the capsaicin patch or the medications that he was on at that time (pregabalin 150 mg ter in die (TID) and methadone 5 mg bis in die (BID)). He decided to discontinue methadone to check if this would have an impact on his pain levels.

**Figure 1:**
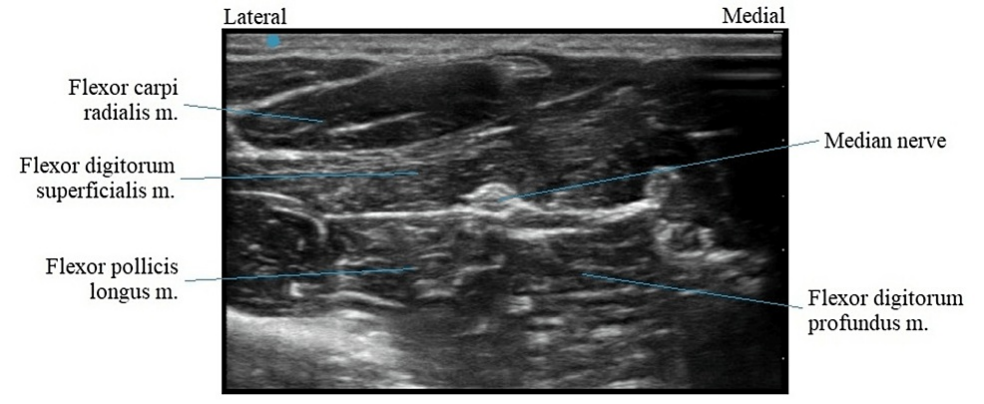
Ultrasonography of the median nerve m.: muscle

**Figure 2:**
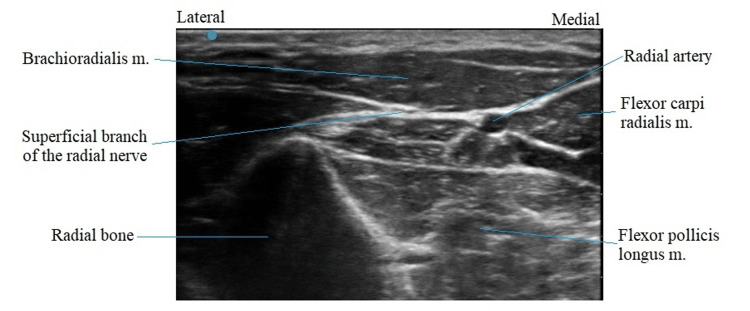
Ultrasonography of the superficial branch of the radial nerve m.: muscle

**Figure 3:**
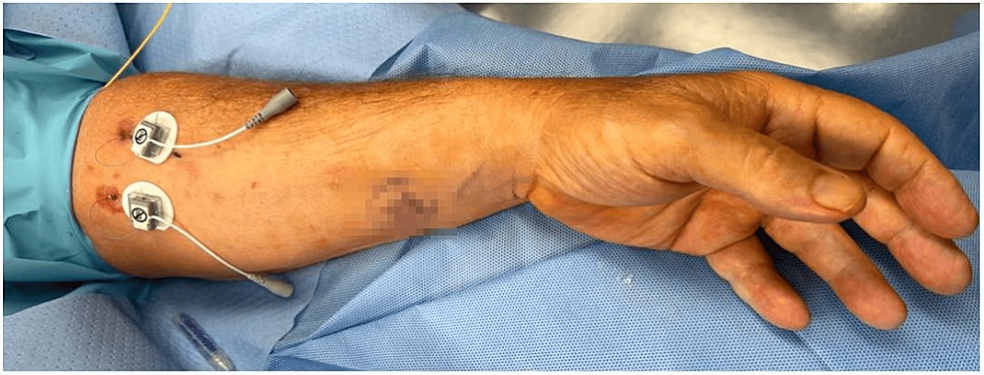
SPRINT electrodes at median and superficial radial nerve

**Figure 4:**
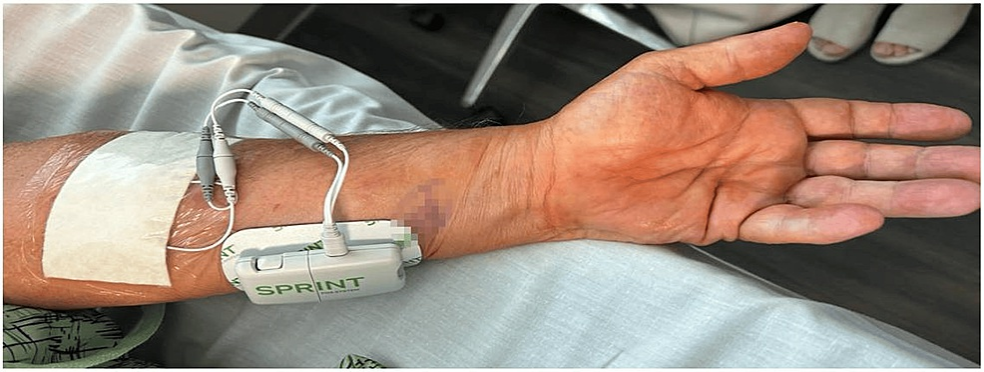
SPRINT PNS implant with external pulse generator PNS: peripheral nerve stimulation

After discussing the long-term treatment options, the patient opted for PNS. The procedure was performed in the following fashion. Pre-procedure cefazolin 2 gram IV was given as antibiotic prophylaxis. He was placed in the supine position, and the left forearm was prepped and draped in a sterile fashion. To anesthetize the skin, 1% lidocaine was used. Using a SPRINT PNS device (SRP Therapeutics, Cleveland, OH), the left median nerve was targeted first under direct ultrasonic guidance (Figure 1). The patient reported sensory stimulation at 30 Hz. The left superficial radial nerve was targeted next (Figure 2). The patient reported a sensory stimulation at 50 Hz. Once the stimulation was confirmed, the MicroLeads (SRP Therapeutics, Cleveland, OH) were implanted and secured with a topical skin adhesive (Figure 3). A median nerve was found at 1.5 cm and, hence, the lead was placed 1.5 cm under the skin. A superficial radial nerve was identified at 0.8 cm, and the lead was placed at 0.8 cm. Programming was done in the recovery room (Figure 4).

Two weeks after the procedure, the patient reported that his pain had reduced to 5/10 from 9/10 on the VAS for pain. However, there was minimal improvement in his quality of life, sleep, and physical function. He found the stimulation of the thumb bothersome, and he was advised to modify the strength of PNS based on his personal preference of balancing the intensity of his chronic neuropathic pain and overstimulation of his thumb. The patient has also discontinued his pregabalin to see whether this would affect his pain.

On the next follow-up, the patient reported continued improvement in his quality of life and sleep, despite cessation of pregabalin and methadone, with average pain of 4/10 and worse pain of 5/10. This persisted for the 62 days that the implants were in place, without any further pain improvement. He can make a fist, which was not possible before this treatment. Residual pain is mostly along the superficial radial nerve distribution and none along the medial nerve. The lingering pain along the superficial radial nerve distribution is likely because of the inability of PNS to overcome considerable neuropathic damage to reconfigure dysfunctional pain transmission signals caused by the inflammatory response against VZV reactivation.

## Discussion

PHN is defined as pain persisting for three or more months after the onset or healing of HZ [6]. PHN is one of the most common complications following shingles. PHN is usually unilateral and dermatomal and overlies the area involved with the shingles rash [7]. Incidence of PHN is approximately 12.8% [8]. The median age for PHN is 65.9 years [8]. HZ virus can affect the peripheral motor and/or sensory nerves. This occurs in 5% to 30% of patients with HZ [9]. Our patient developed PHN in his left upper extremity involving C5-C6 nerve root distribution. 

Literature suggests tricyclic antidepressants (TCAs), gabapentin, pregabalin, and topical lidocaine 5% patch as first-line therapies for PHN [10]. For patients not responding to monotherapy, a combination therapy should be attempted. Other modalities include invasive measures such as intrathecal steroids, sympathetic blockade, and implantable spinal cord stimulators [10].

SCS and PNS are both based on the postulated gate control theory proposed by Melzack et al. [11]. Spinal cord processing acts as a neurologic gate, where networks of nociceptive signals are blocked by intense tactile stimulation from the same area as the pain signal [12]. SCS has been used for the treatment of chronic pain since 1976 [13]. Moreover, it has proven effective in the management of post-herpetic neuralgia [14]. However, it involves placing metal leads, platinum-iridium alloys being the most used electrode material because of its conductivity and biocompatibility, in the spinal dorsal epidural space [15,16]. Additionally, it is associated with rare but serious complications such as paralysis or severe neurological deficits [13].

PNS, on the other hand, stimulates the peripheral nerves directly via a SPRINT PNS device, thereby closing “the gate” and blocking the transition of painful stimuli at the level of the spinal cord [12]. After peripheral nerve stimulator implementation, over 60% of patients had improvement in their symptoms and quality of life [12]. In conjunction with the gate control theory, it has also been proposed that PNS can alter the release of local neurotransmitters that are typically associated with chronic pain leading to neuroplastic effects that modulate pain perception, resulting in sustained pain relief [17].

There are many studies on the use of PNS and its outcome for chronic pain management, but studies are limited to the upper extremities. One secondary retrospective study review done by Huntoon et al. includes 6,100 patients who had SPRINT PNS devices implanted. The most targeted areas were the back and the trunk, lower extremities, and posterior head and neck. This study shows that 71% of patients reported ≥50% pain relief and improved quality of life after 60 days of percutaneous PNS treatment. The total rate of reported adverse events was 6.0%, with the most frequent being skin irritation at 2.6%, followed by painful or uncomfortable stimulation at 1.1%, and other medical events such as swelling or pain at the lead exit site comprising the rest [18]. Additionally, a case report by Kurklinsky et al. describes PHN in the thoracic area, which was treated with subcutaneous PNS and was followed up for 2.5 years [17]. The study showed a pain decrease of up to 80% after subcutaneous PNS. Subcutaneous PNS, typically in place for a 60-day treatment period, can be an effective alternative for patients who are not good candidates for SCS or have failed SCS treatment [19]. Based on these study results, we have opted for PNS to address our patient’s symptoms circumventing the need for a cervical spinal cord stimulator, which is more invasive compared to a PNS.

## Conclusions

To our knowledge, this is the first attempt at treating PHN of the upper extremity with PNS. It has provided a substantial improvement in our patient’s symptoms and seems to be an alternative to SCS in the management of refractory PHN. Future studies need to be conducted to assess and compare the safety profiles and efficacy of these two treatment modalities for refractory PHN.
